# The *ACE2* expression in Sertoli cells and germ cells may cause male reproductive disorder after SARS‐CoV‐2 infection

**DOI:** 10.1111/jcmm.15541

**Published:** 2020-06-28

**Authors:** Qiaoyan Shen, Xia Xiao, Aili Aierken, Wei Yue, Xiaojie Wu, Mingzhi Liao, Jinlian Hua

**Affiliations:** ^1^ College of Veterinary Medicine Shaanxi Centre of Stem Cells Engineering & Technology Northwest A&F University Yangling China; ^2^ College of LifeSciences Northwest A&F University Yangling China

**Keywords:** ACE2, COVID‐19, reproductive disorder, scRNA‐seq, testicular cells

## Abstract

The serious coronavirus disease‐2019 (COVID‐19) was first reported in December 2019 in Wuhan, China. COVID‐19 is an infectious disease caused by severe acute respiratory syndrome‐coronavirus 2 (SARS‐CoV‐2). Angiotensin converting enzyme 2(ACE2) is the cellular receptor for SARS‐CoV‐2. Considering the critical roles of testicular cells for the transmission of genetic information between generations, we analyzed single‐cell RNA‐sequencing (scRNA‐seq) data of adult human testis. The mRNA expression of ACE2 was expressed in both germ cells and somatic cells. Moreover, the positive rate of ACE2 in testes of infertile men was higher than normal, which indicates that SARS‐CoV‐2 may cause reproductive disorders through pathway activated by ACE2 and the men with reproductive disorder may easily to be infected by SARS‐CoV‐2. The expression level of ACE2 was related to the age, and the mid‐aged with higher positive rate than young men testicular cells. Taken together, this research provides a biological background of the potential route for infection of SARS‐CoV‐2 and may enable rapid deciphering male‐related reproductive disorders induced by COVID‐19.

## 1 INTRODUCTION

A newly identified coronavirus, severe acute respiratory syndrome‐coronavirus 2 (SARS‐CoV‐2), has been posing significant threats to public health.^1^ The receptor of coronavirus determines the virus entry into the host cell and constitutes a target for the development of prophylactics and therapeutics.^2^, ^3^ SARS‐CoV‐2 with rapid global sp‐ read was that the SARS‐CoV‐2 binds ACE2 with higher affinity than SARS‐CoV.^4^ Therefore, the analysis of *ACE2* is essential to obtain the route of infection.

The testis is the key of the male reproductive system. Several studies have revealed the expression of ACE2 in testis; but the antibody detection might be subjected to non‐specificity issue.^5^ The recently developed single‐cell RNA sequencing (scRNA‐seq) approaches can effectively delineate cell types and uncover heterogeneity that enables us to specifically detect *ACE2* expression.^6^ Infertility is the most serious type of reproductive disorder, here, we performed to analyse scRNA‐ seq data of testis from five donors with health and eight donors with infertility.^7^, ^8^

## 2 MATERIALS AND METHODS

### 2.1 Public dataset acquisition and processing

Single‐cell datasets (GSE106487, GSE112013) for human testis were obtained from the Gene Expression Omnibus (GEO; https://www.ncbi.nlm.nih.gov/). GSE112013 consists of 6490 single cells and 3002 testicular single cells come from three normal young males.^7^ GSE106487 includes samples from two adult normal males (N), seven obstructive azoospermia males (OA) and one nonobstructive azoospermia males (NOA).^8^ Seurat (version 3.1.3) was used to read two datasets separately and discriminate different cell types. Firstly, datasets were normalized by NormalizeData method. Seurat FindClusters function was used to obtain cell cluster with resolution 0.7 in GSE106487 and 0.4 in GSE112013.

### 2.2 Identification of cell types and gene expression analysis

To obtain potential marker genes, Seurat function FindAllMarkers was used to per‐ form differential expression analysis with the default parameters. UMAP was used for visualization purposes. Besides, Canonical markers for cell clusters were selected and plotted using ggplot2 (version 3.2.1).

According to the previous research articles, SARS‐CoV‐2 tends to attack cells through receptor ACE2, therefore, we defined that *ACE2* positive ratio is the number of *ACE2* positive cells which expression value is larger than 0 within a cluster divided by a cluster total cell number. To clarify the relationship between *ACE2* and regulatory protein, we used RNA and protein network data of *ACE2* through GeneCards (https://www.genecards.org/). The relationship network data were imported into Cytoscape (version 3.7.2), and *ACE2* was highlighted in yellow.

## 3 RESULTS

### 3.1 scRNA‐seq analysis of the human testicular cells

The scRNA‐seq offers new possibilities to address biological and medical questions, the diverse scRNA‐seq protocols and depths showed different efficient for transc‐ riptome quantification, so we performed to two data from different sources for analysis.^7^, ^8^ Group one with mid‐aged, the different donors were labelled for more accurate data analysis with cells coloured based on their donors origin (Figure S1A). 12 different cell types with four types of somatic cells and eight germ cell types were identified (Figures S1B and S2). We also analysed the data from young men for comparison, donors marked with a different colour (Figure S1C). After initial quality controls, 12 different cell types were identified in testis (Figures S1D and S3).

### 3.2 The *ACE2* expression in human testicular cells

**Figure 1:**
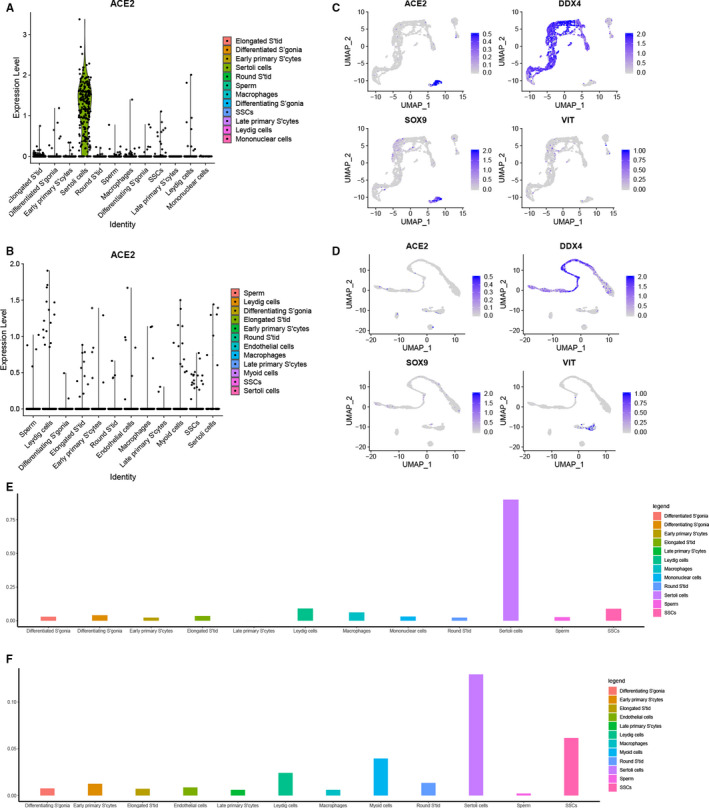
scRNA‐seq analysis of *ACE2* in human testicular cells. A, Expression patterns (violin plot) showing the mRNA expression of *ACE2* in the 12 clusters of the mid‐aged. The Sertoli cells are the highest positive cluster. B, The violin plot of mRNA expression of *ACE2* across all cell types of the young group. C, UMAP plot showing the mRNA expression *ACE2* across clusters of mid‐aged. The Sertoli cells (*SOX9*) are the highest *ACE2*‐positive cells, SSCs with the highest *ACE2* positive rate in Germ cells (*DDX4*), and some Leydig cells (*VIT*) are positive. D, In young group, the *ACE2*‐positive cells shown in the Germ cells, Sertoli cells and Leydig cells. E, The percentage of *ACE2*‐positive cells in different type cells in mid‐aged, Sertoli cells with the highest positive cluster (over 90%). F, Sertoli cells are the highest cluster with *ACE2*‐positive cells in the young group

The mRNA of *ACE2* were expressed in somatic and germ cells (Figure 1A,C). In mid‐aged, about 9% cells with *ACE2* positive in SSCs, and Sertoli cells with higher *ACE2* enrichment (90% of cells; Figure 1E). In young group, the mRNA expression of *ACE2* was still identified in similar cell types (Figure 1B,C,F). Somatic cells are a key component of the testicular microenvironment, which play a significant role in the maintenance of SSCs and spermatogenesis (Figure 1A,B).

### 3.3 The analysis of *ACE2* between healthy and infertility men

**Figure 2:**
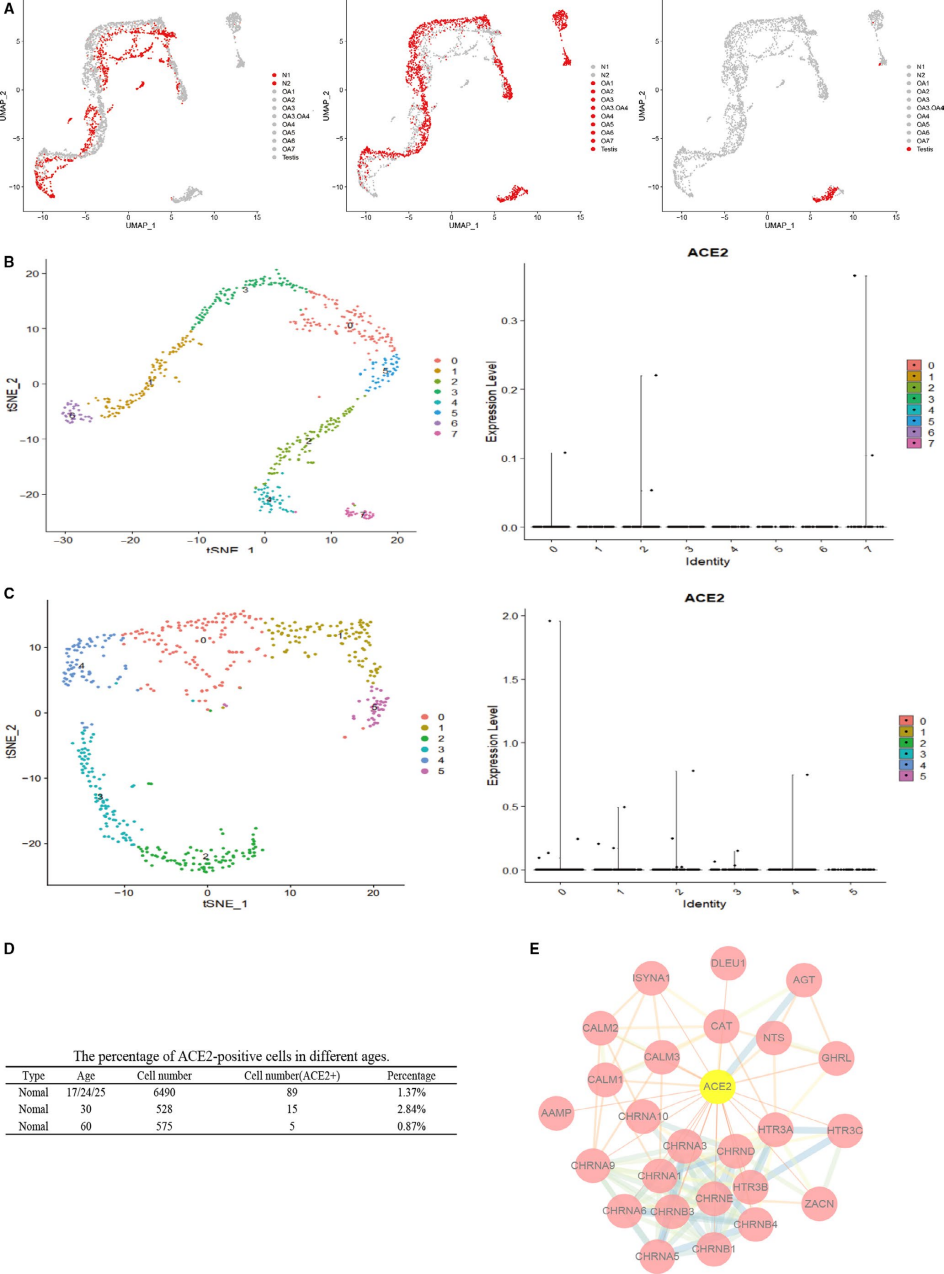
*ACE2* expression in health and male infertility donors. A, UMAP plots showing the characteristics of N (left), OA (middle) and NOA (right) donors. The health are lower than that donors with reproductive dysfunction. B, tSNE plot of testicular cells to visualize cell‐type clusters (60 y old). The violin plot of *ACE2* gene expression across all cell types in testis. C, tSNE plot of testicular cells to visualize cell‐type clusters (30 y old), and violin plot of *ACE2* gene expression across all cell types in testis. D, The percentage of *ACE2*‐positive cells of different ages. In 30‐y‐old, men with the highest *ACE2* positive rate, while about 20 y was relatively low, and the lowest ratio was the 60‐y‐old. E, *ACE2* is interacting with proteins of inflammation‐related HTR3A and tumorigenesis‐related CHRNA1

We noticed that the tendency was similar in two groups, but the specific data were different. The mid‐aged group included some pathological donors, so it was inferred that the expression of *ACE2* is associated with reproductive disorders. To further de‐ termine the characteristic expression of *ACE2*, we analysed the proportion of *ACE2* positive cells in different donors and found that the positive rate of the normal male was lower than that reproductive dysfunction (Figure 2A). The testicular cells of the infected SARS were seriously damaged, IgG was highly expressed in Sertoli cells and germ cells of the patients, and there was also expressed in the Leydig cells, which was similar to the distribution of *ACE2* in the testis.^9^

### 3.4 The mRNA expression of *ACE2* in testis at different ages

SARS‐CoV‐2 showed a various infection rate at different ages.^10^ We analysed the positive cell rate of *ACE2* in testis with healthy men, the highest was 2.84% in 30‐year‐old donor, followed by 20‐year‐old donors (1.37%), and the 60‐year‐old donors were about 0.87% (Figure 2B‐D). Moreover, the online data of GeneCards was analysed to explore potential regulatory mechanism of *ACE2* in testis (Figure 2E). The results showed that *ACE2* has related regulatory effects on 5‐Hydroxytryptamine Receptor 3A (HTR3A) and Cholinergic Receptor Nicotinic Alpha 1 (CHRNA1). *HTR3A* mediated apoptosis indirectly via TNF‐α production by enhancing serotonin release.^11^ *CHRNA1* interacts with inflammation‐related Leucocyte immunoglobulin‐like receptor subfamily B member 3 (L1LRB3). These data suggest the potential mechanism of abnormal function or even damage in patients infected with SARS‐CoV‐2.

## 4 DISCUSSION

ACE2 is a functional receptor for coronaviruses.^4^ In this study, we explored scRNA‐seq data and found that the proportion of *ACE2* positive cells in testis is more than 1%, which is higher than that in lung, indicated that the testis might serve as a high‐risk potential infection organ.^6^ The testicular cell type with inflammation in SARS patients is consistent with the Sertoli cell cluster in our analysis, where *ACE2* expression is abundant.^9^ We noticed that the positive rate of *ACE2* in testis of infertile was higher than normal men, which indicates that SARS‐CoV‐2 may cause reproductive disorders through abnormal activation of ACE2 pathway. One study found that SARS‐CoV‐2 in the semen of some male patients with COVID‐19, further implicating that testis is a portal of infection.^12^ In addition, *ACE2* is widely distributed in SSC, Sertoli cells, and Leydig cells. These findings indicate these cells as possible loci of infection and sexual transmission might be a part of the transmission routes.

SARS‐CoV‐2 had various infection rates in humans at different ages, and men had a higher prevalence than women.^13^ Previous studies have shown that gender was an important factor affecting ACE2 concentrations in plasma.^13^ Therefore, we investi‐ gated the positive rate of *ACE2* in testicular cells of man at different ages. Here, we found that 30‐year‐old men with the highest *ACE2* positive rate and 60‐year‐old men with the lowest ratio in testis. Men had higher mortality than women in the SARS epidemic.^14^ Moreover, SARS‐CoV had a lower lethality rate for 2‐month‐old male mice than 8‐month‐old male mice and relatively higher mortality in middle‐aged mice.^15^ These data suggest that viral infection might be associated with androgen secretion. Different treatment regimens should be appropriate for male and female patients considering androgen levels. Our studies may help with COVID‐19 prevention and control.

## CONFLICT OF INTEREST

The authors of this manuscript have no conflicts of interest to disclose.

## AUTHOR CONTRIBUTIONS

**Qiaoyan Shen:** Conceptualization (lead); data curation (equal); formal analysis (equal); methodology (equal); visualization (equal); writing‐original draft (lead); writing‐review & editing (lead). **Xia Xiao:** Data curation (equal); formal analysis (equal); methodology (equal); software (lead); writing‐original draft (equal); writing‐review & editing (equal). **Aili Aierken:** Writing‐original draft (supporting). **Wei Yue:** Writing‐review & editing (supporting). **Xiaojie Wu:** Writing‐review & editing (supporting). **Mingzhi Liao:** Conceptualization (equal); funding acquisition (lead); project administration (equal); writing‐original draft (equal); writing‐review & editing (equal). **Jinlian Hua:** Funding acquisition (lead); project administration (lead); writing‐original draft (equal); writing‐review & editing (equal).

## Supporting information

Figure S1

Figure S2

Figure S3

## Data Availability

The data that support the findings of this study are available on request from the corresponding author.
