# Supplementary figures and images for: The ACE2 expression in Sertoli cells and germ cells may cause male reproductive disorder after SARS‐CoV‐2 infection
Source: J Cell Mol Med. 2020 Jun 28;24(16):9472–7. doi: 10.1111/jcmm.15541 (PMC7361928; doi:10.1111/jcmm.15541)

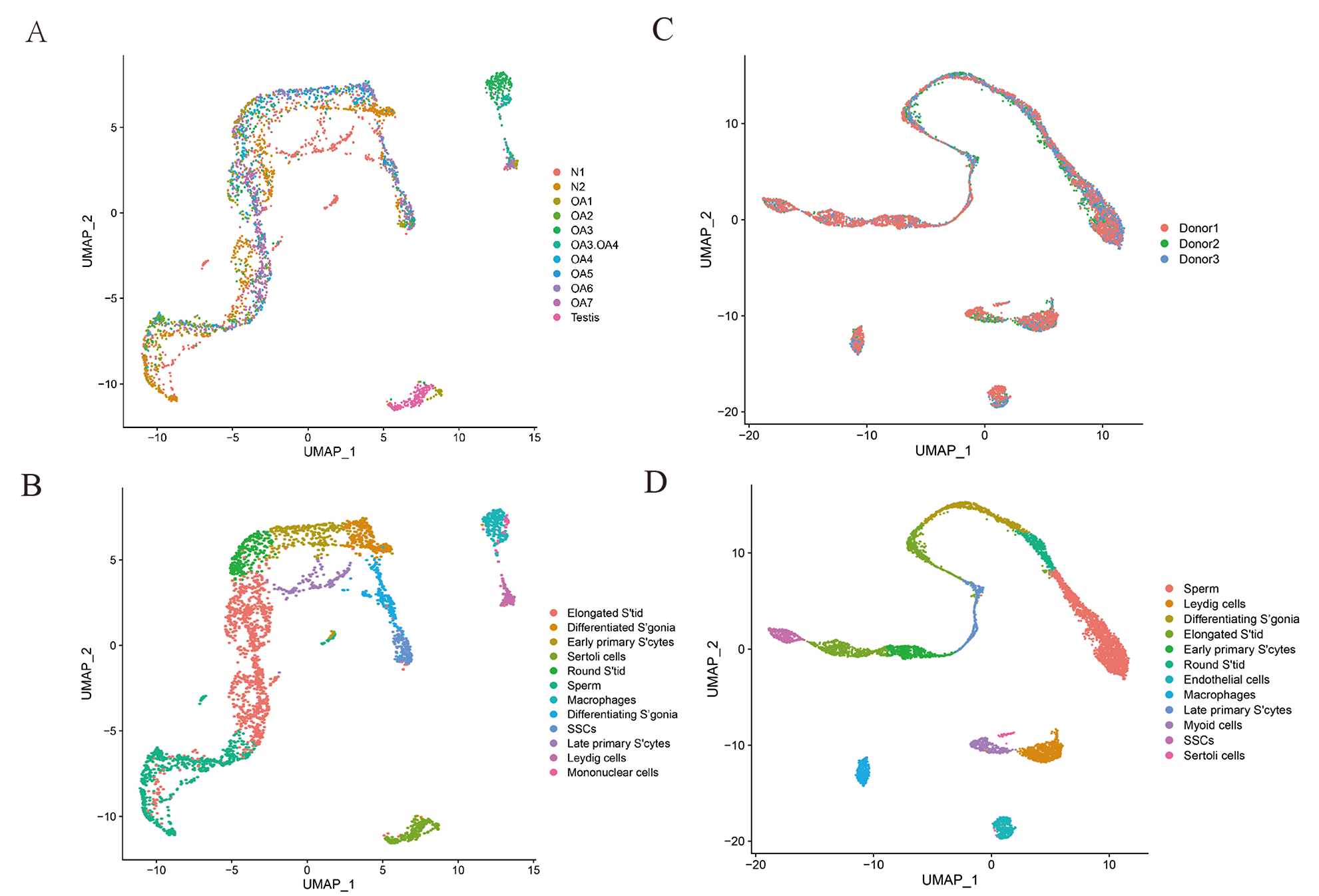

Supplement: Supplementary file 1 — Figure S1 [file JCMM-24-9472-s001.tif]

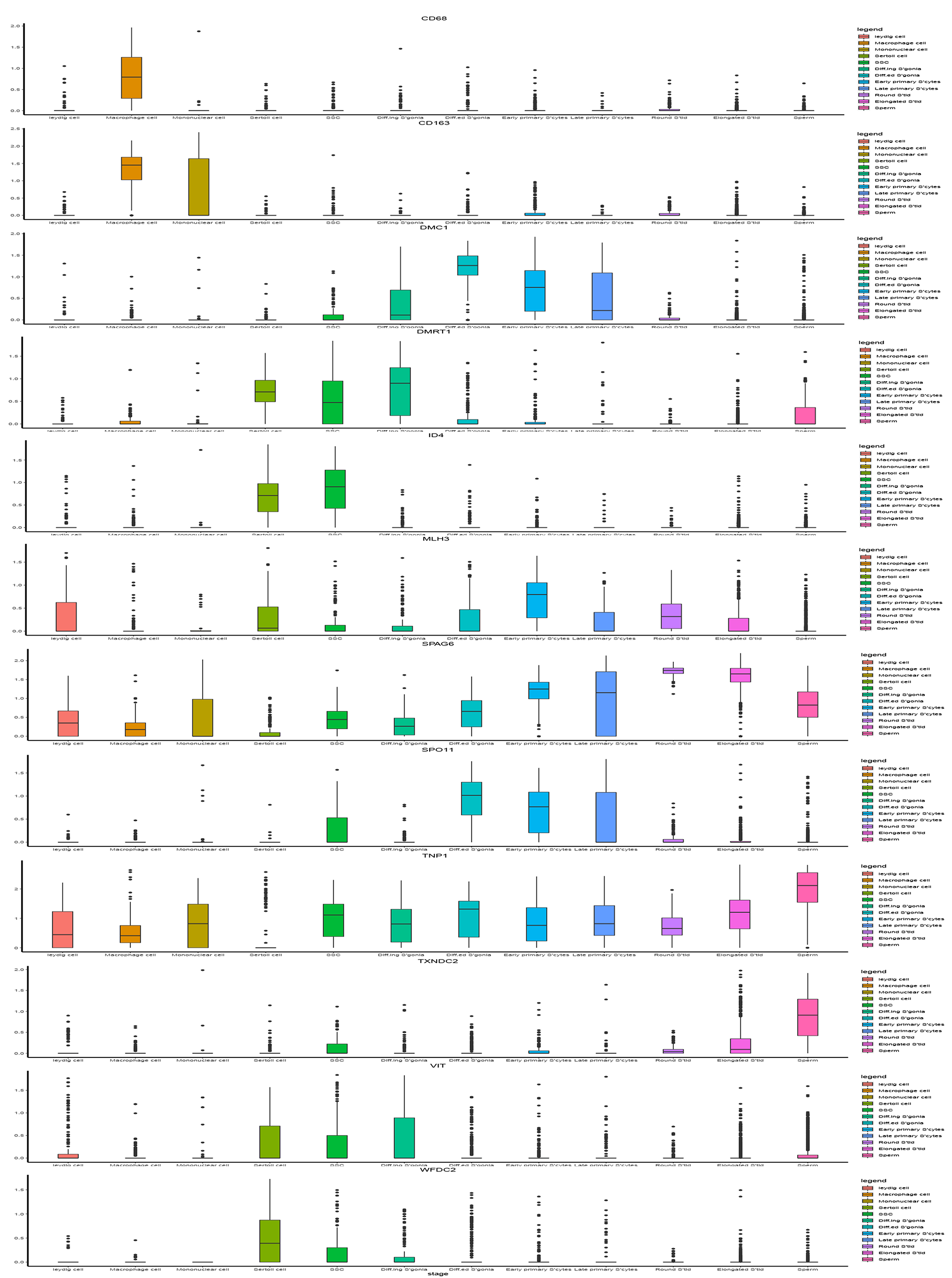

Supplement: Supplementary file 2 — Figure S2 [file JCMM-24-9472-s002.tif]

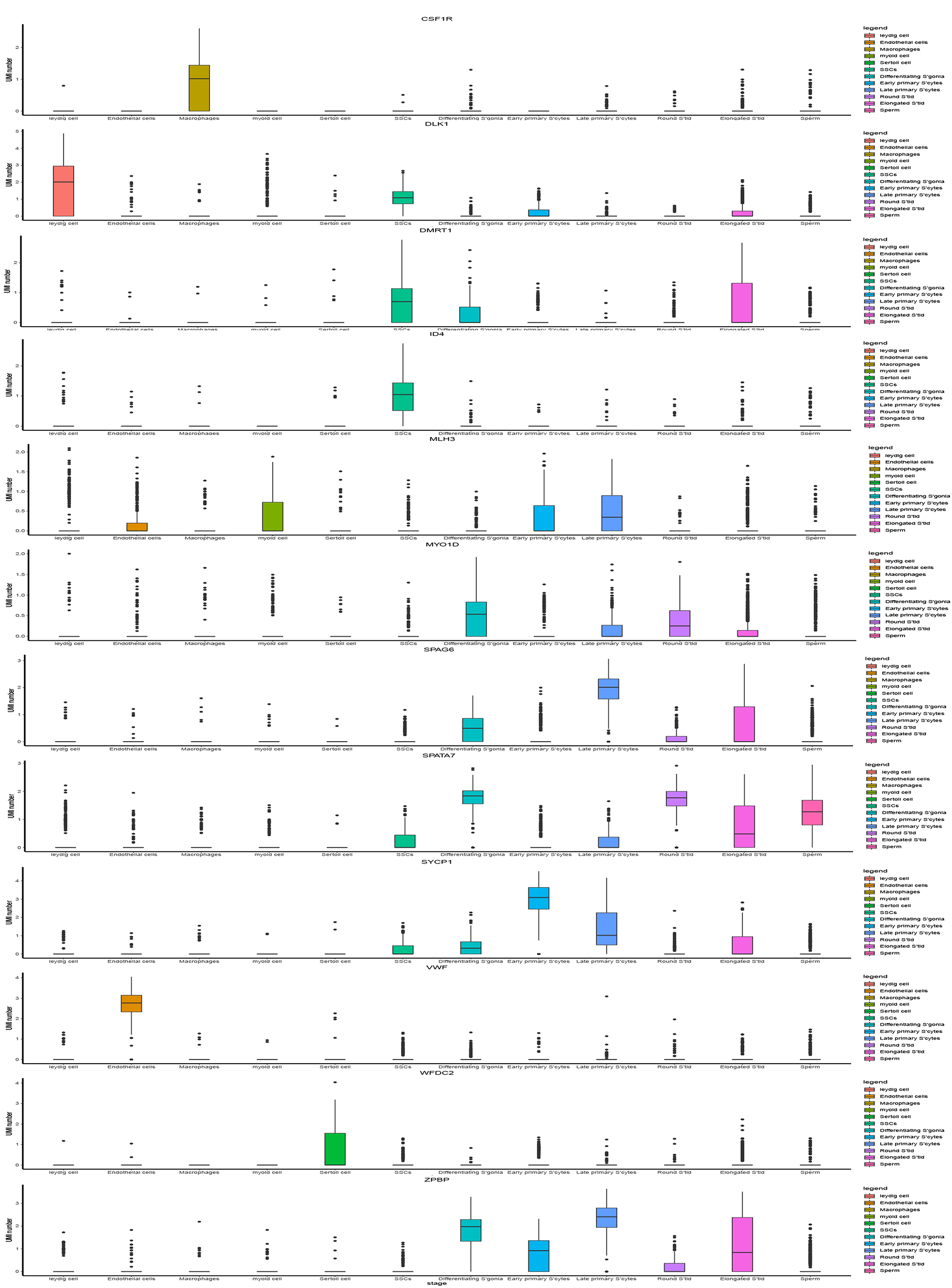

Supplement: Supplementary file 3 — Figure S3 [file JCMM-24-9472-s003.tif]
